# Sex-Based Differences in the Association between Serum Copper and Kidney Function: Evidence from NHANES 2011–2016

**DOI:** 10.3390/ijerph192114086

**Published:** 2022-10-28

**Authors:** Yaxing Nan, Yana Bai

**Affiliations:** 1 College of Earth and Environmental Sciences, Lanzhou University, Lanzhou 730000, China; 2 School of Economics and Management, Gansu University of Chinese Medicine, Lanzhou 730000, China; 3 Department of Epidemiology and Statistics, School of Public Health, Lanzhou University, Lanzhou 730000, China

**Keywords:** copper, trace element, kidney function, glomerular filtration rate (GFR), urine albumin-to-creatinine ratio (UACR)

## Abstract

Epidemiological evidence on the relationship between copper (Cu) and kidney function is rare, and few studies examine the sex differences in this association. We aimed to explore the overall and sex-based relationship between exposure to Cu and biomarkers of kidney function among 4331 participants of the 2011–2016 National Health and Nutrition Examination Survey. Multiple linear regression models were fitted to examine the overall and sex-specific associations between serum Cu and the kidney function indicator-estimated glomerular filtration rate (eGFR) and urinary albumin–creatinine ratio (UACR). Restricted cubic spline models (RCS) stratified by sex were performed to explore the sex-based dose–response associations. Serum Cu in the highest quartile was associated with higher levels of UACR (β = 0.203, 95% CI: 0.100 to 0.306) among overall participants. In males, there was an association of the highest Cu quartile with decreased eGFR (β = −0.023, 95% CI: −0.042 to −0.003) and increased UACR (β = 0.349, 95% CI: 0.171 to 0.527); serum Cu levels also demonstrated a negative nonlinear dose–response association with eGFR and a positive linear dose–response association with UACR in males, whereas females showed a marginally significant nonlinear positive association of eGFR with serum Cu levels. In conclusion, there were sex-specific and dose–response relationships between serum Cu and kidney function indicators. Further prospective and mechanistic studies are warranted.

## 1. Introduction

Chronic kidney disease (CKD) is of increasing global concern, estimated to affect close to 11–13% of the population worldwide, and approximately 15% of the population in the United States (US) [1,2]. The clinical markers eGFR and UACR are frequently used to assess renal function and are both important predictors of the initiation and development of CKD [3]. CKD is characterized by a gradual decline in kidney function and is defined as a reduced estimated glomerular filtration rate (eGFR) lower than 60 mL/min/1.73 m^2^ and/or a urinary albumin-to-creatinine ratio (UACR) above 30 mg/g creatinine for over three months [4,5]. The pathogenesis underlying CKD is complicated and is insufficiently understood. As a critical component that plays an integral role in maintaining total body homeostasis by waste excretion, the kidney is a major target organ of heavy metal exposure [6]. Thus, increasing studies have linked metal exposure to the risk of kidney dysfunction [7,8].

Copper (Cu), one of the most abundant heavy metals, is an essential trace element for the human body [9]. As a catalytic cofactor of enzymes and structural components, Cu is involved in many fundamental and critical physiological processes, including growth, nutrition, and metabolism [10]. However, as a common metal pollutant to the environment, Cu overload may also lead to adverse health effects by catalyzing the production of reactive oxygen species and inducing oxidant-related injury to a wide range of molecules, including lipids and proteins [11,12]. Excessive copper exposure can cause organ toxicity, specifically in the liver and brain [12,13,14]. While exposure to excess Cu exhibited renal toxicity in vitro and in rodents, knowledge obtained from epidemiological studies is limited [12,15]. Two cross-sectional studies on multiple metal exposure have identified urinary Cu levels to be associated with increased odds of CKD or proteinuria [6,16]. However, few studies have focused on the association between serum Cu and the eGFR and UACR, particularly in a sex-specific manner.

Therefore, based on the US National Health and Nutrition Examination Survey (NHANES) between 2011–2016, this study was conducted to explore the relationship between serum Cu and kidney function, as well as the gender differences in this association

## 2. Materials and Methods

### 2.1. Study Population

**Figure A1:**
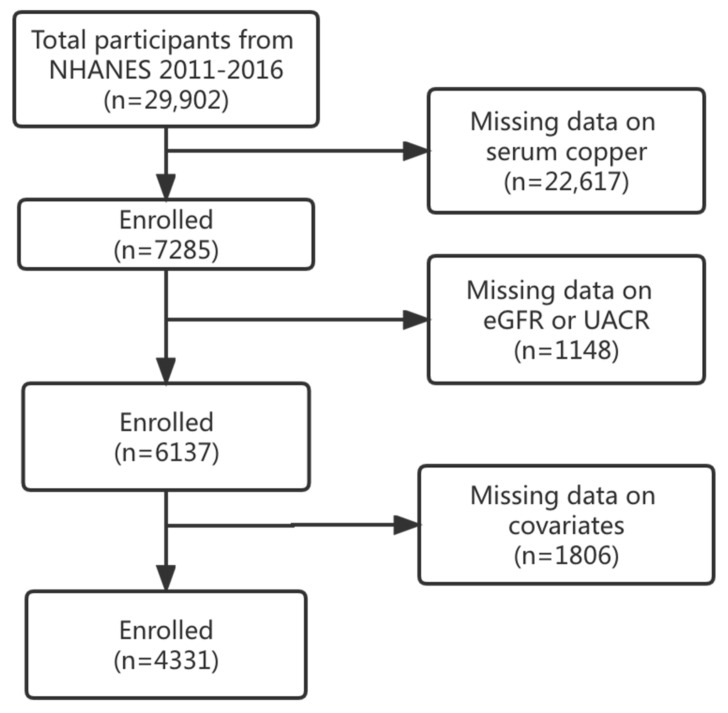
Flow chart of sample selection.

The NHANES, conducted by the National Center for Health Statistics (NCHS), is an ongoing national survey of the health and nutritional status of the general US population, aiming to generate nationally representative estimates [17]. The NHANES study protocol was approved by the NCHS institutional review board, and all participants provided written informed consent. We used the combined six-year dataset derived from the NHANES 2011–2012, 2013–2014, and 2015–2016 cycles (total N = 29,902). Among all the participants, 22,617 with missing values on serum Cu, and 1148 with missing values on the eGFR or UACR were excluded from analyses. Those for whom the covariate data were missing were further excluded (N = 1806). Finally, 4331 participants were enrolled in the final analyses (Figure A1).

### 2.2. Measurement of Serum Cu

The serum Cu levels were detected using inductively coupled plasma dynamic reaction cell mass spectrometry (ICP-DRC-MS). The lower limit of detection for serum Cu was 2.5 (μg/dL). Detailed information on the detection methods and procedures is available online (https://wwwn.cdc.gov/nchs/nhanes/default.aspx, accessed on 3 May 2022).

### 2.3. Outcome Assessment

The outcomes for the present study were kidney function-related indicators: the eGFR and UACR, measured as continuous variables. The eGFR was measured using the CKD-EPI Creatinine Equation (2021), using serum creatinine, age, and sex [18]. Serum creatinine was analyzed by the Jaffe rate method with BeckmanUniCel DxC800 Synchron (Beckman, Fullerton, CA, USA). The UACR was calculated as the urine albumin/creatinine ratio. Urine albumin was evaluated from a spot sample via a fluorescent immunoassay (SequoiaTurner 450 Digital Fluorometer, Block Scientific, Holbrook, NY, USA) [19].

### 2.4. Covariates

Covariates were included based on previous knowledge of risk factors for kidney dysfunction [20,21,22], including age (<20, 20–39, 40–59, ≥60 years), sex, education (less than high school, high school or equivalent, and college or above), race, poverty–income ratio (<1.3, 1.3–3.5, or >3.5), body mass index (BMI: ≥30.0 kg/m^2^, 25.0 to <30.0 kg/m^2^, 18.5 to <25.0 kg/m^2^, or <18.5 kg/m^2^), smoking (never, current, former), alcohol drinking (yes or no), and hypertension and diabetes status (yes or no). Hypertension was defined as either self-reported hypertension diagnosis, use of antihypertension medication, measured systolic blood pressure ≥ 140 mmHg, or measured diastolic blood pressure ≥ 90 mmHg [22]. Diabetes was ascertained from either self-reported physician diagnosis, fasting glucose > 7 mmol/L, HbA1c ≥ 6.5%, or self-reported use of insulin or oral hypoglycemic medication [14].

### 2.5. Statistical Analysis

Characteristics of the study population are presented as numbers and percentages stratified by sex. Values of continuous variables were described as medians and interquartile range [IQR]. For categorical variables, the frequency and percentage were presented. Serum Cu concentrations were natural log transformed to reduce skewness or categorized into quartiles, from lowest (Q1) to highest (Q4). Owing to their skewed distribution, the eGFR and UACR were also transformed into natural logarithms before analysis. The multiple linear regression models were fitted to examine the relationship between serum Cu and kidney function indicators, including the eGFR and UACR. Tests for a linear trend across quartiles (Q) of serum Cu levels were conducted, and the values of the P-trend were calculated by including the median of each quartile (ln-transformed Cu concentrations) as a continuous variable [23]. Models were adjusted for age, sex, race, BMI, educational attainment, PIR, smoking status, alcohol status, and history of hypertension and diabetes; all the covariates were categorical variables, as indicated in Section 2.4. To explore the potential sex heterogeneities, we performed the same analyses in the subgroup analyses by sex. In order to detect the sex-specific dose–response relationships of serum Cu and kidney function, we fitted sex-stratified restricted cubic spline models (RCS). For this, the R package *‘rms’* was used to estimate the Poverall and Pnonlinear. A nonlinear dose–response relationship was demonstrated if Poverall and Pnonlinear were both below 0.05. A linear dose–response relationship would be suggested if just Poverall was less than 0.05. Since Zinc (Zn) and Cu often biologically interact [24], we conducted sensitivity analyses by further adjusting for the levels of ln-transformed serum Zn in the models to demonstrate how interactions between the two metals might change the magnitude of associations. Two-sided *p*-value of <0.05 was considered statistically significant. All data were analyzed by R version 4.0.4 software (R Foundation for Statistical Computing, Vienna, Austria).

## 3. Results

### 3.1. General Characteristics

The general characteristics of the 4331 participants (2166 men and 2165 women) are shown in Table 1. Significant sex differences were identified with regard to kidney function indicators: women had higher eGFR and UACR compared to men (*p* < 0.05). Higher levels of serum Cu were also detected in women. Furthermore, more women received a college education; men had higher proportions in Non-Hispanic Blacks and smaller proportions in Other Hispanics; men also had a higher prevalence of diabetes, and higher alcohol and smoking rates, and more women were obese, whereas more men were overweight (all *p* < 0.05).

### 3.2. Overall and Sex-Specific Associations between Serum Cu and Kidney Function

**Figure 1:**
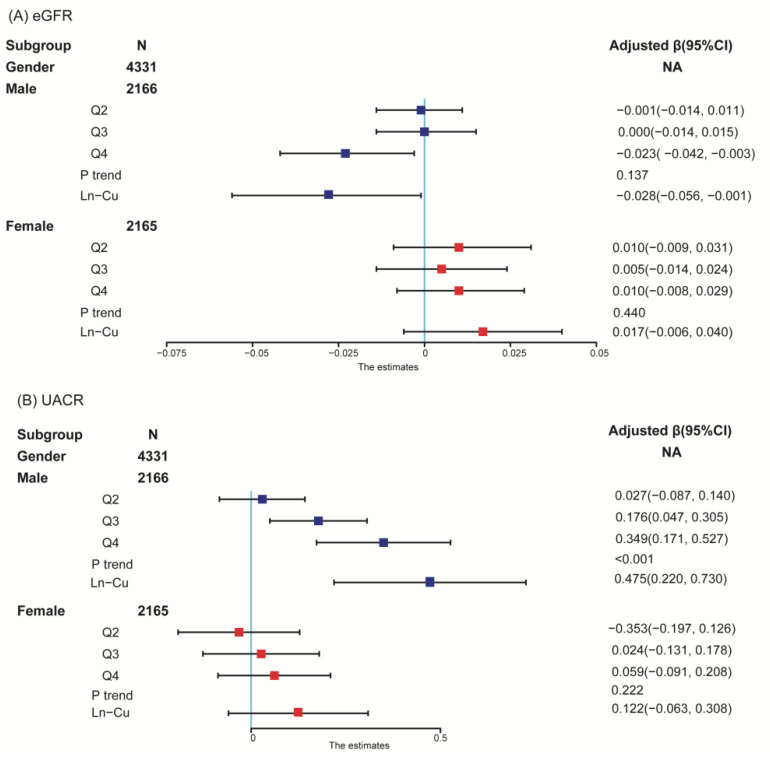
Sex-specific association between serum Cu and kidney function. (**A**) eGFR; (**B**) UACR. Blue and red boxes represent males and females respectively. Models adjusted for age, race, BMI, educational attainment, PIR, alcohol drinking, smoking status, hypertension, and diabetes. The points represent the adjusted β, and the lines represent its 95% confidence interval (95% CI).

Presented in Table 2 are the overall associations between serum Cu and kidney function indicators derived from multiple linear regression models. The beta values (β) represent the regression coefficients. Among the overall population, continuous serum Cu levels were positively associated with UACR (β = 0.297, 95% CI:0.145 to 0.448), serum Cu levels at Q4 had a higher UACR compared with Q1 (β = 0.203, 95% CI: 0.100 to 0.306). Figure 1 presents the sex-specific association of serum Cu with kidney parameters. For men, serum Cu at Q4 was associated with a decreased eGFR and increased UACR (β = −0.023, 95% CI:−0.056 to −0.001 for eGFR; β = 0.349, 95% CI: 0.171 to 0.527 for UACR); each unit of ln-transformed serum Cu concentrations predicted a −0.028 (CI:−0.056, −0.001) decrease of ln-transformed eGFR, and a 0.475 (CI: 0.220, 0.730) increase of ln-transformed UACR. However, neither kidney function indicator was associated with serum Cu levels in females. The results of sensitivity analyses by further adjusting for serum Zn levels were consistent with the above findings (Table A1).

### 3.3. Dose–Response Associations

**Figure 2:**
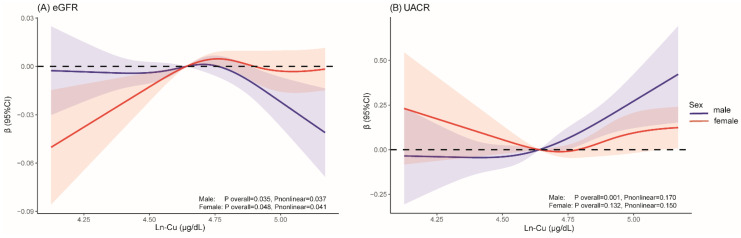
Sex-specific restricted cubic spline (RCS) analysis of dose–response relationship between serum Cu levels and kidney function. (**A**) eGFR; (**B**) UACR. The lines with shading represent adjusted β [95% confidence interval (CI)] based on RCS. Models adjusted for age, race, BMI, educational attainment, PIR, alcohol drinking, smoking status, hypertension, and diabetes.

The dose–response associations between serum Cu and kidney function based on the RCS analyses are shown in Figure 2. Males demonstrated a negative nonlinear relationship between serum Cu and the eGFR (*p* overall = 0.035, *p* nonlinear = 0.037) and a positive linear relationship with UACR (*p* overall = 0.001, *p* nonlinear = 0.170). Females showed a positive nonlinear relationship of eGFR (*p* overall = 0.048, *p* nonlinear = 0.041).

## 4. Discussion

In the present study, we examined the overall and sex-specific association between serum Cu and kidney function indicators and examined sex differences in this association. High levels of serum Cu may elevate UACR levels among all participants and males, and correlate with a decline in eGFR in males. In the dose–response analyses, males also demonstrated a negative nonlinear association with eGFR and a positive linear association with UACR with serum Cu concentrations, whereas females exhibited a nonlinear positive association with eGFR with serum Cu at low concentrations.

The serum Cu concentrations for both sexes in this study were within the recommended reference range, between 63.5–158.9 µg/dL [25], but the concentrations of serum Cu were significantly higher in women (127 µg/dL) than in men (104 µg/dL) (*p* < 0.05). Other studies also found that women were exposed to higher levels of Cu than men [26,27]. Hormonal differences between men and women may help explain the differential distribution of Cu between the sexes. Endogenous hormones may affect Cu metabolism at the cellular and whole-body levels [28]. Animal studies suggest higher levels of estrogen may enhance the production of ceruloplasmin, which is an important Cu-transporting protein, and hence increase the absorption of Cu and elevate serum Cu levels [28,29,30].

Cu is an essential trace element necessary for various metabolic functions in the human body [11]. There is an extensive literature documenting the potential hazard of impaired Cu homeostasis, which is implicated in various cardiovascular, gastrointestinal, and neurological diseases and metabolic disorders [9,31,32]. Previous epidemiological studies revealed a link between Cu exposure and kidney impairment. Tsai’s cross-sectional study indicated high urine Cu associated with proteinuria and an eGFR of <60 mL/min/1.73 m^2^ [6]. Another cross-sectional study in rural China identified urine Cu significantly correlated with higher odds of abnormal eGFR [16]. In the present study, high serum Cu was significantly associated with increased UACR in males and the overall participants, and with a decrease in eGFR in males. As Cu and Zn may compete through the absorption stage and are biologically connected [24], we conducted sensitivity analyses by accounting for the interactions of serum Cu and Zn. After further adjustment for serum Zn levels, the results of our study remained consistent, suggesting the robustness of our findings. The mechanism(s) by which high Cu induces kidney impairment is not well-understood. The kidney is a crucial component for metabolism and is particularly susceptible to oxidative stress [22]. Existing evidence based on animal studies suggests that excess Cu may trigger oxidative stress and cause lipid membrane peroxidation [12]. Thus, oxidative stress may have played a key mechanistic role in explaining Cu-induced nephrotoxicity. The mechanism studies, however, were based on animal models; hence, further study into the Cu-related mechanism of renal dysfunction is needed.

To our knowledge, few studies examined the sex-based differences in the association between Cu exposure and kidney function. The present study reveals that, although the concentrations of serum Cu were lower in men, they were more prone to renal injury in relation to Cu exposure compared to women. The female participants even conversely showed a marginally significant association of higher eGFR with increasing serum Cu. Despite previous literature rarely addressing the sex-differential nephrotoxicity of Cu, other risk factors for kidney dysfunction were found to be related with gender in previous research, and sex hormone responsiveness might also account for this sex disparity [33,34]. Estrogens may protect against renal injury through their antifibrotic, antiapoptotic, and antioxidant effects, while testosterone may be pro-inflammatory [34]. A meta-analysis of 68 studies concluded that men were more prone to rapid renal impairment among nondiabetic CKD patients compared with women [35]. Rodent models also revealed that male rats exhibited a more rapid decline in kidney function with the effect of aging [34]. Another animal experiment on rats found that age-matched female rats demonstrated a significantly lower extent of kidney dysfunction in experimentally induced CKD caused by chronic nitric oxide inhibition [36]. Sex hormones were considered to have a major impact on mediating these observed sex disparities [34]. Whether or not sex hormones are involved in the potential sex differences of Cu-induced kidney function decline deserves further research.

The strengths of our analyses include the application of the NHANES dataset, which is based on a large nationally representative survey with rigorous and standardized procedures of data collection. In addition, after accounting for a variety of confounding variables, including serum Zn levels, our results remained significant. However, some limitations in our study should be acknowledged. First, the cross-sectional nature of the study does not allow for causality inference. Second, despite multiple confounders having been adjusted, residual confounding due to unknown or unmeasured variables cannot be completely ruled out.

## 5. Conclusions

The present study suggested sex-specific associations of serum Cu with kidney function: in males, serum Cu levels had a nonlinear relationship with a lower eGFR and a linear relationship with a higher UACR, whereas females showed a marginally significant nonlinear association of a higher eGFR with increasing serum Cu. Further research is required to confirm the findings in prospective and mechanistic studies.

## Figures and Tables

**Table 1:** Characteristics of study population (NHANES, 2011–2016).

	Overall	Males	Females	
Variable ^a^	N = 4331	N = 2166	N = 2165	*p*-Value ^b^
Age groups, years				0.394
<20	147 (3.39%)	67 (3.09%)	80 (3.70%)	
20–39	1476 (34.1%)	756 (34.9%)	720 (33.3%)	
40–59	1358 (31.4%)	662 (30.6%)	696 (32.1%)	
≥60	1350 (31.2%)	681 (31.4%)	669 (30.9%)	
Race/ethnicity				0.038
Non-Hispanic White	1739 (40.2%)	886 (40.9%)	853 (39.4%)	
Non-Hispanic Black	891 (20.6%)	462 (21.3%)	429 (19.8%)	
Other Hispanic	467 (10.8%)	206 (9.51%)	261 (12.1%)	
Other race	1234 (28.5%)	612 (28.3%)	622 (28.7%)	
PIR				0.371
Low	1429 (33.0%)	694 (32.0%)	735 (33.9%)	
Middle	1591 (36.7%)	801 (37.0%)	790 (36.5%)	
High	1311 (30.3%)	671 (31.0%)	640 (29.6%)	
Educational attainment				0.015
Less than high school	932 (21.5%)	486 (22.4%)	446 (20.6%)	
High school or equivalent	1001 (23.1%)	528 (24.4%)	473 (21.8%)	
College or above	2398 (55.4%)	1152 (53.2%)	1246 (57.6%)	
BMI, kg/m^2^				<0.001
Underweight	66 (1.54%)	23 (1.07%)	43 (2.01%)	
Normal weight	1227 (28.6%)	607 (28.2%)	620 (28.9%)	
Overweight	1391 (32.4%)	806 (37.4%)	585 (27.3%)	
Obese	1613 (37.5%)	718 (33.3%)	895 (41.8%)	
Smoking status				<0.001
Never	2485 (57.4%)	1053 (48.6%)	1432 (66.1%)	
Ever	1014 (23.4%)	631 (29.1%)	383 (17.7%)	
Current	832 (19.2%)	482 (22.3%)	350 (16.2%)	
Alcohol drinking status				<0.001
No	1225 (28.3%)	378 (17.5%)	847 (39.1%)	
Yes	3103 (71.6%)	1787 (82.5%)	1316 (60.8%)	
Diabetes				0.030
No	3712 (85.7%)	1831 (84.5%)	1881 (86.9%)	
Yes	619 (14.3%)	335 (15.5%)	284 (13.1%)	
Hypertension				0.260
No	2756 (63.6%)	1360 (62.8%)	1396 (64.5%)	
Yes	1575 (36.4%)	806 (37.2%)	769 (35.5%)	
Serum Cu, μg/dL	114 [98.7;133]	104 [91.8;117]	127 [110;147]	<0.001
eGFR, ml/min per 1.73 m^2^	103 [90.5;115]	102 [90.2;113]	104 [90.9;117]	<0.001
UACR, mg/g	7.08 [4.69;13.3]	6.16 [4.12;11.9]	8.00 [5.40;14.7]	<0.001

Abbreviations: NHANES: National Health and Nutrition Examination Survey; Cu: copper; BMI: body mass index; eGFR: estimated glomerular filtration rate; UACR, urine albumin-to-creatinine ratio; PIR: poverty–income ratio. ^a^ Continuous values were presented as median (IQR), and categorical variables were shown as numbers (percentage). ^b^ *p*-values for difference between males and females, compared by Wilcoxon rank-sum test for skewed continuous variables, and chi- squared test for categorical variables, *p* ≤ 0.05 identifies statistical significance.

**Table 2:** Overall association of serum Cu with kidney function indicators.

Serum Cu (μg/dL)	eGFR (Ln Transformed)		UACR (Ln Transformed)	
	β (95% CI)	*p*-Value	β (95% CI)	*p*-Value
Overall				
Q1	0.00(Reference)		0.00(Reference)	
Q2	0.003(−0.007, 0.014)	0.537	0.049(−0.042, 0.134)	0.293
Q3	0.002(−0.009, 0.013)	0.702	0.145(0.050, 0.240)	0.003
Q4	0.001(−0.010, 0.013)	0.805	0.203(0.100, 0.306)	<0.001
*p* trend	0.882		<0.001	
Ln-Cu	0.006(−0.012, 0.022)	0.533	0.297(0.145, 0.448)	<0.001

Abbreviations: Cu: copper; eGFR: estimated glomerular filtration rate; UACR: urine albumin-to-creatinine ratio; CI: confidence interval; Q: quartile. The values of β are presented as the adjusted coefficients obtained from multiple linear regression models. Models adjusted for age, sex, race, BMI, educational attainment, PIR, alcohol drinking, smoking status, hypertension, and diabetes.

## Data Availability

Publicly available datasets were analyzed in this study. The data can be found at https://wwwn.cdc.gov/nchs/nhanes/ (accessed on 3 May 2022).

## Appendix A

**Table A1:** Sensitivity analyses for the association of serum Cu with kidney function indicators after further adjusting for serum Zn.

Serum Cu (μg/dL)	eGFR (Ln Transformed)		UACR (Ln Transformed)	
	β (95% CI)	*p*	β (95% CI)	*p*
Overall				
Q1	0.00(Reference)		0.00(Reference)	
Q2	0.002(−0.008, 0.013)	0.649	0.051(−0.040, 0.142)	0.269
Q3	0.000(−0.011, 0.011)	0.969	0.149(0.054, 0.245)	0.002
Q4	−0.001(−0.012, 0.011)	0.925	0.209(0.106, 0.312)	<0.001
*p* trend	0.839		<0.001	
Ln-Cu	0.003(−0.014, 0.021)	0.720	0.303(0.151, 0.455)	<0.001
Male				
Q1	0.00(Reference)		0.00(Reference)	
Q2	−0.002 (−0.015, 0.010)	0.698	0.028	0.628
Q3	−0.002(−0.016, 0.012)	0.799	0.179	0.007
Q4	−0.003(−0.045, 0.006)	0.011	0.352	<0.001
*p* trend	0.066		<0.001	
Ln-Cu	−0.034(−0.062, −0.006)	0.017	0.483(0.226, 0.739)	<0.001
Female				
Q1	0.00(Reference)		0.00(Reference)	
Q2	0.010(−0.010, 0.030)	0.332	−0.034(−0.195, 0.128)	0.682
Q3	0.003(−0.015, 0.022)	0.718	0.026(−0.129, 0.181)	0.742
Q4	0.008(−0.011, 0.026)	0.408	0.063(−0.087, 0.213)	0.413
*p* trend	0.628		0.158	
Ln-Cu	0.015(−0.008, 0.038)	0.201	0.125(−0.060, 0.311)	0.185

Abbreviations: Cu: copper; eGFR: estimated glomerular filtration rate; UACR: urine albumin-to-creatinine ratio; CI: confidence interval. Models adjusted for age, sex, race, BMI, educational attainment, PIR, alcohol drinking, smoking status, hypertension, diabetes, and serum zinc levels.
